# Investigating and defining outcomes of suprapatellar versus infrapatellar intramedullary nailing of tibial shaft fractures: a protocol for a pilot randomised controlled trial

**DOI:** 10.1186/s40814-022-01057-5

**Published:** 2022-05-26

**Authors:** Simon Thwaites, Dominic Thewlis, Kelly Hall, Mark Rickman

**Affiliations:** 1 Centre for Orthopaedic & Trauma Research, The University of Adelaide, Adelaide, SA Australia; 2 School of Public Health, The University of Adelaide, Adelaide, SA Australia; 3 Department of Orthopaedics & Trauma, Royal Adelaide Hospital, Adelaide, SA Australia

**Keywords:** Tibial shaft, Fracture, Intramedullary nailing, Suprapatellar, Infrapatellar

## Abstract

**Background:**

Anterior knee pain is often reported following intramedullary nailing of tibial shaft fractures. The aetiology remains unclear, but the surgical approach may play an important role. To date, no biomechanically validated method exists to assess patient outcomes specific to anterior knee pain in this cohort. The central aims of this study are to (1) evaluate the feasibility of a full-scale randomised controlled trial (RCT) investigating the influence of surgical approach on intramedullary nailing of tibial shaft fractures (suprapatellar versus infrapatellar nailing), (2) explore differences in clinical outcomes between the approaches, and (3) explore the development of a biomechanically validated methodology for assessing post-operative anterior knee pain and knee function specific to intramedullary nailing of tibial shaft fractures.

**Methods:**

This pilot study will follow a prospective randomised controlled design at the Royal Adelaide Hospital and The Queen Elizabeth Hospital (South Australia). This study aims to recruit 60 patients between 18 and 60 years old who will be randomly assigned to either the suprapatellar or infrapatellar approach following a decision for intramedullary surgical fixation by the treating surgeon. All nails in this study will be Stryker T2 Alpha nails. Patients will undergo standard radiograph, magnetic resonance imaging, and clinical assessments in-line with their standard operative care, and complete a number of patient-reported and performance-based outcome measures. Performance-based outcome measures will be assessed utilising three-dimensional motion capture techniques. Follow-up time points are 3, 6, 12, and 18 months. Feasibility outcomes include ability to meet enrolment and retention metrics, compliance with all questionnaires and assessment procedures, and the occurrence of any adverse events. The primary clinical outcome is the incidence of anterior knee pain at 12 months after surgery.

**Discussion:**

This study will establish the feasibility and inform the design of a large-scale RCT. Evaluation of all clinical data and patient outcomes will lead to the development of a new tool for assessing patient outcomes in this cohort. Limitations of the study include an unpredictable enrolment rate and loss to follow-up, small sample size, and the unknown ability of three-dimensional motion analysis to pick up the effects of anterior knee pain after tibial nailing.

**Trial registration:**

This trial was prospectively registered on the 7 February 2020 on ANZCTR, ACTRN12620000109909.

## Introduction

### Background and rationale

Tibial shaft fractures are the most common long bone fracture [1]. Intramedullary (IM) nailing—the insertion of a surgical nail along the medullary cavity—is commonly used to stabilise these fractures as it enables stable fixation with minimal soft-tissue damage [2]. IM nailing approaches are generally categorised by the nail entry site: infrapatellar nailing (IPN) is performed transtendinous, or by lateral or medial parapatellar approaches, with the knee in a flexed or hyper-flexed position [3, 4]; conversely, suprapatellar nailing (SPN) is an intra-articular technique allowing the knee to remain in near full extension [5]. The promising results of SPN, especially for proximal third fractures, involved in 5–11% of all cases [6], have led some surgeons to adopt SPN as their standard technique [7], and some authors have recommended SPN for treating all fractures of the tibial shaft [8]. However, there is no conclusive evidence to inform surgical decision making on whether an optimal IM nailing technique exists. The choice of approach is typically based on surgeon experience first, and secondarily the fracture pattern. The absence of biomechanically validated in vivo outcome measures specific to this cohort has led to conflicting studies reporting significant differences between SPN and IPN, while other studies have reported no difference in patient satisfaction [9].

One well-documented disadvantage of IM nailing is long-term anterior knee pain (AKP), occurring in 10% [10] to 86% of cases [11]. AKP post-SPN has been reported as non-existent [5], significantly lower than IPN [8], and not different to IPN [12–15]. These differences may in part be explained by differing study designs and the range of scoring systems used being developed for various other knee pathologies [16–19]. AKP can severely influence long-term activities of daily living [20–22], of which, kneeling causes the most severe knee pain [23], and exacerbates existing pain in 60% of cases [24]. This has potentially significant effects for occupations that require kneeling, as well as some religious activities. The incidence of knee pain resulting from kneeling varies [11, 23, 25, 26] but is reported to be as high as 91.8% [27]. Further, 26 [28] to 50% [27] of patients are unable to kneel at all. Two different forms of kneeling have been described: upright and flexed [29, 30]. This results in different anatomical structures contacting the ground [30] which is likely to influence the incidence of AKP. In order to assess kneeling ability, it is important to consider both forms [31]. To date, there have been four randomised, prospective trials looking at SPN versus IPN [8, 14, 32, 33]; only one study [33] assessed kneeling ability but failed to differentiate between kneeling modalities. No research has explored how biomechanical changes in kneeling might relate to AKP after IM nailing.

Using existing patient-reported outcome measures (PROMs), studies have showed that SPN has similar [2, 23] or better [34] functional outcomes when compared to IPN, yet outcomes based solely from PROMs may not be truly representative. A combination of PROMs and performance-based outcome measures (PBOMS) is required to capture patient recovery adequately. Subjective PROMs do not capture information about the mechanics of the tasks being assessed and have not been biomechanically validated for this patient group. Changes in objectively measured gait biomechanics have been evaluated with patient-perceived outcomes following total knee arthroplasty [35, 36], but no studies have attempted to identify important gait- or task-specific parameters associated with tibial shaft fracture patients treated with IM nailing. The correlation of subjective, self-reported assessments of function and pain with the biomechanics of these activities may provide valuable insight into causes of AKP and restricted functional ability.

This pilot RCT will help to establish the basis for a future large-scale RCT and will explore the outcomes of SPN versus IPN through analyses of the biomechanics of different tasks and the evaluation of these findings with AKP and function questionnaires. Important surgical outcomes, such as tibial alignment, fracture union, and intra-articular damage, will also be captured in this study. It is anticipated that the development of biomechanically validated outcome measures used in a full-scale RCT will enable new guidelines for the treatment of tibial shaft fractures to be developed, as well as novel outcome measures specific to this patient group.

## Objectives

### Primary objective

The primary objective of this study is to determine the feasibility of a full-scale RCT, the aim of which is to investigate differences in patient outcomes between the surgical management of tibial shaft fractures treated with suprapatellar versus infrapatellar intramedullary nailing approaches.

### Secondary objectives

We aim to compare the effect of SPN versus IPN with the incidence of AKP and knee function at 3, 6, 12, and 18 months follow-up, utilising a series of patient-reported outcomes and laboratory-based assessments.

### Tertiary objectives

The tertiary objective of the study is to explore the development of novel three-dimensional (3-D) motion capture based biomechanical outcome measures for assessing AKP and knee function after tibial fracture surgery. This will be achieved by evaluating objectively measured biomechanical outcomes and gait patterns against patient-reported outcome measures of knee pain and function.

Additional objectives include comparing the effect of SPN versus IPN on surgical outcomes including: tibial alignment, rotational profile, intra-articular damage, and time to union.

## Methods and analysis

This protocol was developed in accordance with the Standard Protocol Items: Recommendations for Interventional Trials guidelines and the Consolidated Standards of Reporting Trials 2010 statement: extension to randomised pilot and feasibility trials [37–39].

### Trial design

This study is designed as a prospective, parallel group pilot RCT aiming to compare the results of SPN versus IPN for tibial shaft fractures during the first 18 months after surgery. Randomisation will be computer-generated, restricted by stratification, and will be carried out in a 1:1 manner. Randomisation will be stratified by age (< 40/≥ 40) and patient gender.

For every tibial shaft fracture patient enrolled in the RCT, we aim to recruit a healthy, case-matched volunteer from the general population to generate a normative reference dataset. The healthy cohort of volunteers will be case-matched by considering age, sex, height, and body mass, and screened for eligibility over the phone.

### Patient and public involvement

Patients and public were not involved in the design, conduct, or reporting of this study.

### Eligibility

Patients between 18 and 60 years of age will be consented for the study via the surgical team after their admission to the Royal Adelaide Hospital or The Queen Elizabeth Hospital for the treatment of tibial shaft fracture.

### Inclusion criteria

- 18–60 years old at the time of inclusion;
- Unilateral, extra-articular, tibial shaft fractures;
- Intramedullary fixation is the preferred treatment as determined by the treating orthopaedic surgeon.

### Exclusion criteria

- Unable to write or read English;
- Unable to understand spoken English;
- Unable to give informed consent;
- Fractures involving the tibial metaphysis (fracture location and limited weight bearing after surgery may affect the outcomes being measured);
- Other major trauma to the lower limbs (except for ipsilateral fibular fractures);
- American Society of Anesthesiologists classification of 3 or more at the time of inclusion;
- Significant pre-existing mobility problems as defined by either:
  - A low [40] (less than seven) New Mobility Score [41] (NMS), or
  - A score of less than two for any individual NMS item.

### Randomisation and blinding

Consenting patients will be randomised into either SPN or IPN groups by a member of the surgical team through revealing allocation cards inside concealed envelopes. A randomly permuted blocks schedule was created with two treatment arms (labelled A and B) with equal allocation over four strata (males under 40, females under 40, males 40 and over, females 40 and over). Two block sizes (2 and 4) were used and allocated in equal proportions. A unique, non-informative, 3-digit study subject identifier was generated for each treatment allocation. Fifty allocations were generated per stratum with a total of 200 allocations. This process was replicated for each study site resulting in two unique schedules. The randomisation schema was generated by a University statistician using Stata v15.1 (StataCorp LP, College Station, TX, USA) and the user-written Stata package ralloc [42]. The concealment of allocation cards was performed by researchers outside of the surgical team who will enrol patients and reveal the allocations. Due to the nature of the surgical intervention, patients, surgeons, and researchers will not be blinded to the randomisation allocation as participants will acquire scars at different sites.

### Standard treatment pathway

Patients enrolled into this study will follow standard operative care in line with each institution’s routine clinical practice, the only exception being the surgical approach as directed by the randomisation outcome.

### Allocated interventions

A total of 60 patients with tibial shaft fractures will receive one of two intramedullary nailing interventions:

1. Infrapatellar nail entry: access to the tibia in all IPN approaches will be standardised using the medial parapatellar approach, as is the department standard
2. Suprapatellar nail entry: access to the tibia in all SPN approaches will be standardised using a midline quadriceps incision

All nails in this study will be Stryker T2 Alpha nails (Stryker Corporation, Kalamazoo, MI, USA). Both interventions will be performed by consultant surgeons or senior registrars in training familiar with the procedures. Participants will receive identical preoperative and postoperative treatment in both groups according to the standard protocol of the treatment centre.

### Participant flow timeline

**Fig. 1:**
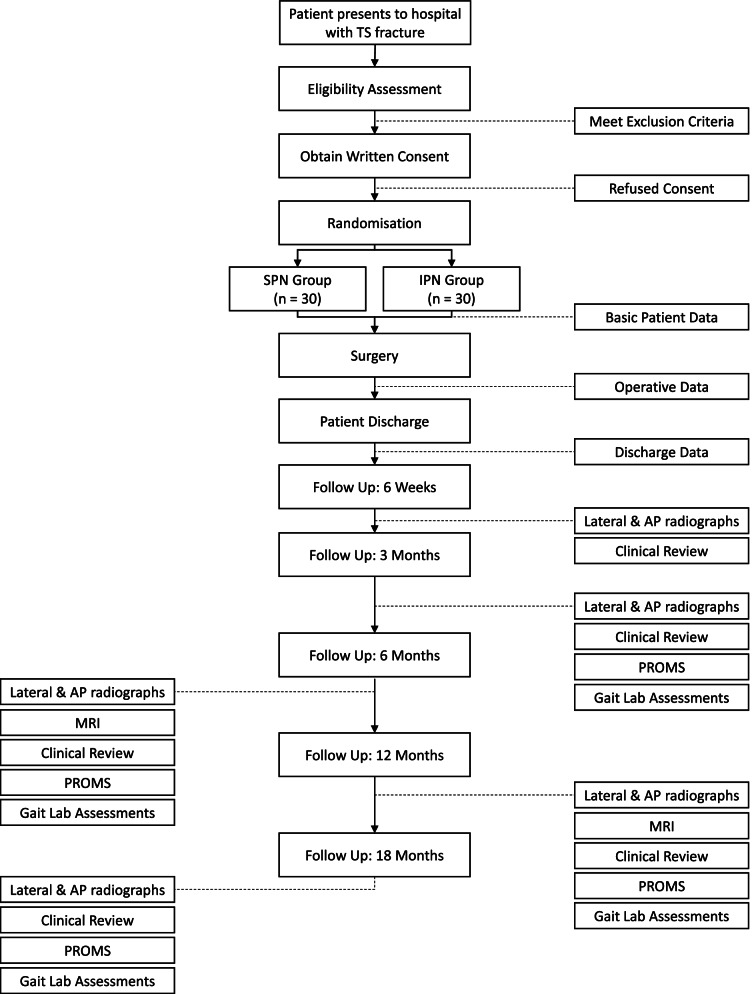
Patient flow diagram. TS, tibial shaft; SPN, suprapatellar nailing; IPN, infrapatellar nailing; AP, anteroposterior; PROMS, patient-reported outcome measures; MRI, magnetic resonance imaging

The timeline for participant involvement in the trial is detailed in Fig. 1. Following randomisation, baseline patient assessment will be completed using an online form (note: this data is not affected by the randomisation allocation). Surgery will proceed at the earliest available opportunity in line with each hospital’s routine clinical practice. Operative information will be recorded using an online form. Recruitment, medical, and surgical data collection will occur at the Royal Adelaide Hospital and The Queen Elizabeth Hospital. Follow-up appointments will be scheduled such that medical imaging, clinical reviews, and gait laboratory assessments coincide. The first clinical review will occur at the treating institution, with all subsequent appointments at the Clinical Research Facility at The University of Adelaide. At each follow-up, patients will present for medical imaging at a diagnostic imaging practice (lateral and anteroposterior radiographs at all follow-ups; magnetic resonance imaging (MRI) at 6 and 12 months); followed by clinical examination with an orthopaedic and trauma specialist; finally, PROMS will be collected using an online form and gait laboratory assessments conducted at the Clinical Research Facility, Adelaide Health and Medical Sciences Building (all follow-ups except 6 weeks). At the conclusion of the gait laboratory assessments, participants will be provided a wrist-worn activity monitor to be returned after 7 days with a reply-paid envelope.

### Participant retention

In order to maximise participant retention, patients will be instructed on the importance of attending all follow-up time-points. All appointments will be made in order to minimise the time-burden on the participants, i.e. scheduling imaging, clinical reviews, and gait laboratory sessions consecutively. Patients will be contacted primarily by phone and email, or mailed letters if contact is unsuccessful. Patients will be reminded of all follow-up sessions.

## Outcomes

### Feasibility outcomes

The primary objective of this study is to determine the feasibility of a larger scale RCT. Feasibility of a larger scale RCT will be determined by assessing the enrolment and retention rates, compliance with questionnaires and assessment procedures, and the occurrence of any adverse events. In the occurrence of any adverse events, the causality of the event in relation to the intervention will be assessed according to the National Health and Medical Research Council guidelines [43].

### Primary clinical outcome measure

The primary clinical outcome measure for the pilot RCT is the incidence of AKP at 12-months follow-up. To assess AKP, participants will be asked to answer the following questions:

1. Do you experience any pain in the knee of the leg that was operated on? Yes/No

2. Was this pain present only after surgery and not beforehand? Yes/No

3. Is this pain located over the front of your knee? Yes/No

If patients answer yes to all questions, their pain will be regarded as postoperative AKP.

### Secondary clinical outcome measures

#### Perioperative outcome measures

We will record operative time (minutes from first incision to closure), blood loss (millilitres), and the time and total radiation dosage used during fluoroscopy (millisieverts). Regarding fluoroscopy, any variations from confounders will be recorded and reported.

#### Postoperative outcome measures

Additional objectives of the study include comparing the effect of surgical outcomes; postoperative outcome measures include:

- Tibial alignment from post-operative lateral and anteroposterior radiographs (all follow-up time-points),
- Time to union as indicated on post-operative radiographs,
- Rotational profile from MRI (6 and 12 months follow-up),
- Intra-articular damage from MRI (6 and 12 months follow-up),
- Subsequent surgeries and complication rate (e.g. compartment syndrome, surgical site infection, malunion, nonunion, screw penetration in the tibiofibular joints),
- Fracture location will also be recorded as this may influence outcomes.

#### Clinical assessment

At each follow-up, a clinician will assess lower extremity function including a general clinical assessment of tenderness, swelling, and range of motion, and record the Knee Society Score [44] (KSS). The KSS is a widely used system to score the function of the knee, comprising two parts: (1) the Knee Score (KS-KS) and (2) the Function Score (KS-FS). The scores range from 0 to 100; a higher score is equivalent to a better outcome. The Minimal Clinically Important Difference (MCID) for the KS-KS ranges from 5.3–5.9 points and for the KS-FS from 6.1–6.4 points [45].

#### Patient reported outcome measures

PROMS will be captured electronically at 3, 6, 12, and 18 months follow-up.

##### Knee injury and osteoarthritis outcome score [16] (KOOS)

The KOOS has been validated for osteoarthritis [16] and for knee arthroplasty [46]. A normalised score between 0 and 100 (0 representing extreme knee problems and 100 representing no knee problems) for each of the five subscales is calculated. The MCID for the KOOS has not been assessed. Importantly, the KOOS contains a question relating to kneeling.

##### KOOS patellofemoral subscale [47] (KOOS-PF)

The KOOS-PF contains 11 items that generate a normalised score from 0 to 100, similar to the KOOS. The subscale was developed for AKP/patellofemoral pain and/or patellofemoral osteoarthritis. The KOOS-PF is suitable to be used in conjunction with the KOOS [47]. The MCID for the KOOS-PF is 11.8 [47].

##### Functional joint Score-12 knee [48] (FJS-12 knee)

The FJS-12 Knee contains 12 items scored from 1 to 5. The raw score is normalised to 0–100, where a high score indicates a good outcome. The FJS-12 Knee displays low ceiling effects in more active and younger patient groups [49]. The FJS-12 Knee has been validated for anterior cruciate ligament reconstruction [49] and knee arthroplasty [50].

##### Quality of life (via EuroQol 5-dimension 5-level (EQ-5D-5L) [51])

The EQ-5D-5L is a standard instrument used to assess general health outcomes, comprising the EQ-5D descriptive system and the EQ visual analogue scale (EQ VAS). The descriptive system comprises 5 dimensions each with 5 levels. The EQ VAS is scored from 0 to 100. EQ-5D-5L provides stronger validity evidence than the EQ-5D-3L for osteoarthritis [52] and has been previously used in IM nail studies [53–55].

##### Visual analogue scale [56] (VAS)

The VAS is a longitudinal scale ranging from 0 to 10 centimetres, zero indicating no pain, and ten indicating the worst pain imaginable. Patients will be asked to score their usual pain for the last week. The MCID for VAS scores is 2 points or 2 cm [56]. VAS is suitable to be administered electronically [57–60].

##### Pain Catastrophizing scale [61] (PCS)

The PCS is a 13-item questionnaire scored from 0 to 4 that provides a valid index of catastrophizing of pain. The total score ranges from 0 to 52. A higher score is associated with more catastrophic thinking. There is no MCID available for the PCS.

#### Covariates

##### New mobility score [41] (NMS)

The NMS contains three items pertaining to patients’ ability to walk indoors, outdoors, and go shopping. Each item is scored from 0 to 3, resulting in a score from 0 to 9, where nine indicates a high level of mobility.

##### Medication

Patients will be asked to detail any current medication at all follow-ups.

##### Rehabilitation

Patients will be asked to detail the number of physiotherapy appointments they have recently attended at all follow-ups.

##### Return to work

As time from surgery as indicated at any of the follow-up time-points. We will also record any changes to occupation or working capacity resulting from the leg injury or from AKP.

#### Performance based outcome measures

PBOMS will be conducted at 3, 6, 12, and 18 months follow-up. Three-dimensional (3-D) kinematic data will be captured with a 10-camera motion capture system (Vicon Motion Systems Ltd, Oxford, UK, 100 Hz) in line with standardised protocols [62]. Ground reaction forces will be captured using two in-ground force platforms (AMTI Optima, Watertown, MA, USA, 2000 Hz). Lower extremity muscle activity will be measured using passive surface electromyography electrodes (Delsys, Boston, MA, USA, 2000 Hz). A custom Matlab user-interface (MathWorks, Natick, MA, USA) and Vicon Nexus 2.9 (Vicon Motion Systems Ltd, Oxford, UK) will be used for data capture. Musculoskeletal modelling using OpenSim [63] will simulate gait parameters including (but not limited to) knee kinematics, knee joint contact forces, and knee muscle forces.

In addition to walking gait at self-selected speed, participants will conduct a series of performance-based functional tests to generate data related to AKP, knee function, and thigh muscle performance, including:

- Maximum isometric thigh muscle strength recorded with a MicroFET2 digital handheld dynamometer (HOGGAN Scientific, Salt Lake City, UT, USA) [64];
- Kneeling ability assessed using:
  - Aberdeen Weight-Bearing Test (Knee) [33] (AWT-K) in upright and flexed positions. The AWT-K tests for anterior knee discomfort using the ratio of weight distribution between both legs during kneeling
  - Kneeling range of motion reaching tasks similar to previously described in literature [65]
  - The effect of cushioning using Mechanix Wear 700 Series knee pads (Mechanix Wear, Valencia, CA, USA)
  - The anatomic structures in contact with the ground during these kneeling assessments will be determined using a combination of eight force sensitive resistors (Delsys, Boston, MA, USA) arranged about the patella similar to previously described in literature [65]
- ‘Timed up and go’ test [66];
- Single-legged anterior reach test [67];
- Timed seated leg extension hold;
- Squatting;
- Anterior single-legged, single hop-for-distance test [67];
- Step up/over test [68] and step down test [69].

Twenty-four-hour physical activity patterns will be recorded for 7 days following gait-lab sessions using a wrist-worn activity monitor (GeneActiv Original, Activinsights Ltd, Kimbolton, UK, 100 Hz). Patients will be asked to keep a sleep log to differentiate sedentary and sleep time. Physical activity captured using wrist-worn accelerometers on either wrist strongly correlates with devices worn around the waist but are less obtrusive [70].

An overview of all data collection at all time-points is presented in Table 1.

**Table 1:**
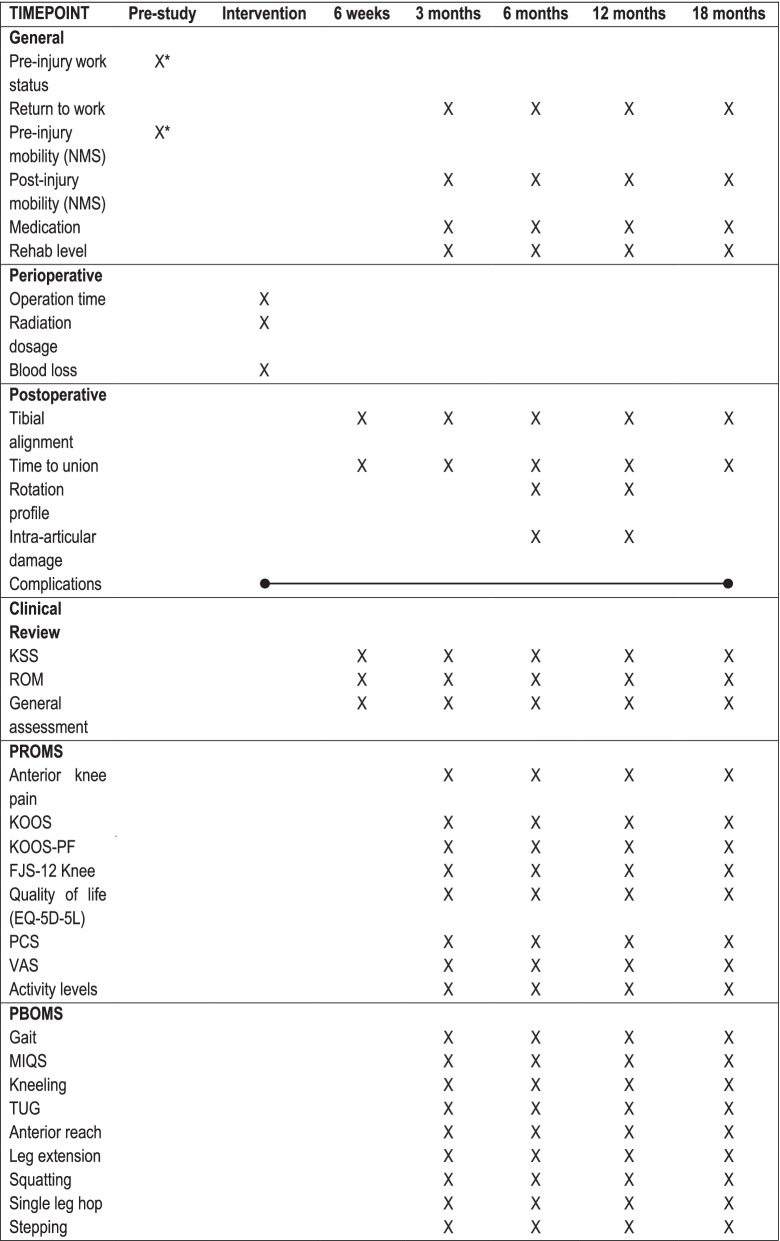
Overview of data collection from intervention to 18-months follow-up

*Captured retrospectively after discharge

*NMS* New Mobility Score, *KSS* Knee Society Score, *ROM* range of motion, *PROMS* patient reported outcome measures, *KOOS* Knee Injury and Osteoarthritis Outcome Score, *KOOS-PF* KOOS patellofemoral subscale, *FJS-12 Knee* Functional Joint Score-Knee, *EQ-5D-5L* EuroQoL 5-Dimension 5-Level, *PCS* Pain Catastrophizing Scale, *VAS* visual analogue scale, *PBOMS* performance based outcome measures, *MIQS* maximum isometric quadriceps strength, *TUG* timed up-and-go

### Exploratory clinical outcomes

The tertiary objective of this study is the development of novel outcome measures specific to IM nailing of tibial shaft fractures from a series of performance-based and patient-reported outcomes captured in the pilot RCT. The successful development of the new tool using a combination of PROMs and PBOMs will be assessed on its ability to differentiate participants with and without AKP.

### Protocol deviations

Patients that undergo nail removal will be asked to be followed up for an additional 18 months after nail removal. Indications for nail removal may include patient preference, knee pain, and infection [71].

### Data management

All questionnaire data, except for the KSS and sleep logs, are captured electronically. The KSS is captured on paper by the treating clinician at the clinical reviews; the sleep logs are captured on paper by the participants. Both paper forms are converted into electronic format through completion of electronic forms that are designed to not allow manual text entry (i.e. only radio button selection) in order to ensure data accuracy during transfer. All data is then stored electronically on password-protected shared drives and backed up weekly to a password-protected folder on the University of Adelaide’s network. All hard-copy questionnaire and consent forms are stored in locked compactus storage requiring key-card access. Only investigators will have access to the data.

### Sample size

This study has been designed as a pilot study to determine the feasibility of a large-scale RCT. Therefore, no sample size calculation was performed. Based on the number of fractures in a year at the recruitment sites, we aim to recruit a total of 60 patients; allowing for some loss to follow-up, should enable 25 patients to complete follow-up assessments from each group (SPN and IPN), which is in-line with sample size recommendations for pilot studies [72].

### Statistical analysis

Enrolment rates will be calculated as the ratio between the number of enrolled participants and the total number of eligible patients presenting at the enrolment sites. Retention rates will be determined by the total number of enrolled participants completing their 18-month follow-up appointment. Compliance with questionnaire and assessment procedures will be assessed by analysing the completion rates of all questionnaires and assessments (e.g. radiographs and MRI). All adverse events will be recorded and the total number and impact of all adverse events rated on a single 7-point Likert scale ranging from 0 to 6, with 6 indicating no impact from adverse events. See Table 2 for a description of feasibility outcome metrics and progression criteria based on the traffic light system [73]:

**Table 2:** Feasibility outcome metrics and traffic light system progression criteria

Outcome	Red: major revision prior to full trial	Amber: continue to full trial with changes	Green: continue to full trial without changes
Enrolment rate (target: 60 enrolled after 18 months)	< 60 % of eligible patients enrolled	60–80% of eligible patients enrolled	> 80% of eligible patients enrolled
Retention rate	< 70% complete 18-month follow-up	70–80% complete 18-month follow-up	> 80% complete 18-month follow-up
Questionnaire and assessment compliance	< 80% completion of all questionnaires and assessments	80–90% completion of all questionnaires and assessments	> 90% completion of all questionnaires and assessments
Adverse events	Score of < 3 on a 7-point Likert scale indicating total impact of all adverse events	Score of 3 or 4 on a 7-point Likert scale	Score of ≥ 5 on a 7-point Likert scale.

Green: continue to full trial without changes

Amber: continue to full trial with changes to the protocol as deemed necessary by the Trial Steering Committee relating to enrolment rate, retention rate, questionnaire and assessment compliance, and the occurrence of any adverse events

Red: do not continue to full trial. Major revisions to the protocol are required. Trial Steering Committee to determine whether the study warrants continuing even with major revisions

As this is a pilot study, any analyses related to the clinical outcomes should be treated with caution and viewed as exploratory. All clinical outcome measures will be assessed descriptively (means and standard deviations for normally distributed continuous variables; medians and interquartile ranges for non-normal continuous variables; frequencies and percentages for categorical variables). Missing data will be tabulated.

Logistic regression models will be used to determine the association between surgical approach and AKP 12 months post-surgery. Models will be adjusted for age and gender with hospital included as a fixed effect. The results will be reported as odds ratios with 95% confidence intervals.

Secondary clinical outcomes will be analysed using linear regression models for continuous outcomes or generalised linear models for count or categorical outcomes. Generalised estimating equations will be used to account for correlation due to repeated measures over time, and a time-by-surgery interaction term will be included to test for differences in the relationship between surgical approach and outcomes over time. If assumptions about distributions are not met (e.g. non-normally distributed continuous outcomes) alternative approaches will be explored as appropriate, including transformations or non-parametric modelling.

To assess differences between cases and matched healthy controls, logistic regression models will be used to determine the association between treatment (surgery versus no surgery) and AKP 12 months post-surgery. Models will be adjusted for the matching variables age, gender, and body mass index, with hospital also included as a fixed effect.

To determine novel predictors of functional outcomes, logistic regression prediction models will be used to assess the discriminatory power of PROMs and PBOMs at assessing AKP and other functional measures. Estimates of discrimination (area under the curve, sensitivity, and specificity) will be used to rank combinations of outcomes in ability to predict AKP. Principal component analysis (PCA) will be explored as a method of data reduction to allow information from a larger number of variables to be included in the predictive model. If PCA is successful, a predictive model will be fitted using an appropriate number of principal components (determined using the proportion of variance explained as well as number of events in the dataset) as predictors.

For all outcomes, effect estimates and 95% confidence intervals will be reported to express uncertainty about the estimated effects.

## Discussion

This protocol describes the design of pilot randomised controlled trial investigating the influence of SPN vs IPN on AKP after intramedullary nailing of tibial shaft fractures. In the last 10 years, studies have shown SPN increases accuracy of the nail entry point to the tibia [12], improves insertion angles [74], and is associated with good alignment rates [5, 13]. IPN has been associated with higher rates of malalignment [6, 75–77] in part because the flexed knee required for IPN can lead to extension of the proximal fracture fragment, making reduction difficult [75]. Additionally, the relatively easier reduction and positioning provided by SPN is associated with reduced fluoroscopy time [8, 78, 79]. The major drawback of SPN is concerns regarding the articular approach [14, 80–82] and permanently decreased quadriceps strength resulting from nail entry [80]. However, MRI and arthroscopic assessment of the patellofemoral joint after SPN and IPN suggests minimal risk [5, 14], and several anatomical studies show similar articular damage between approaches [83–85]. The influence of nail removal (if it occurs) should also be considered, as removal is always performed via an infrapatellar incision, regardless of the initial approach [86].

The aetiology of AKP remains unclear [86], yet the choice of approach may play an important role: patients undergoing SPN may be more prone to loss of quadriceps function and damage to intra-articular structures; whereas IPN patients may suffer from increased likelihood of damage to the patellar tendon, fat pad, and saphenous nerve. A meta-analysis of 20 papers found an average of 47% of patients reported AKP at an average follow-up of 2 years, although how the AKP was assessed was not detailed in the review [87]. Common modalities used to assess knee pain after tibial shaft nailing include the Lysholm Test [17], Oxford Knee Score [18], and the Kujala Score [19]; none of these tools are specific to AKP after tibial nailing. Further, simply asking whether any pain exists is frequently used [25, 88–91], but the use of such a binary measure is problematic for future studies as large numbers of participants are required in order to power the study, e.g. if there is truly no difference between IPN and SPN treatment (assuming absence of AKP is 53% in both groups), then 1080 patients are required to be 90% sure that the limits of a two-sided 90% confidence interval will exclude a difference between groups of more than 10%.

An important factor when considering PROMs, such as kneeling ability, is the potential disparity between patients’ perception and their actual ability to perform the activity. Though comparable studies for IM nailing are lacking, the inability of PROMs to detail patient recovery sufficiently after total knee arthroplasty has been well described [92–95]. Hassaballa et al. found 37% of patients believed they had the ability to kneel following IM nailing, but 81% could kneel upon instruction [96]. The current study, to the authors’ knowledge, is the first study aiming to collect both patient-reported and 3-D motion capture based outcomes for this cohort.

The results of this pilot study will help inform the design of a large-scale RCT. Progression criteria, using the traffic light system, relating to the feasibility outcome metrics presented in Table 2, will inform the definitive trial: if all metrics are green, continue to the full trial; if any metrics are amber, continue to the full trial with appropriate modifications to the protocol as determined by the Trial Steering Committee; if any metrics are red, do not continue to the full trial as major revisions to the trial protocol are required before continuing, or the trial should not continue. Further, this study will explore the development of novel, biomechanically validated outcome measures for AKP and knee function and aid in the development of a new tool for assessing AKP in this cohort. Any clinical outcomes between approaches should be treated with caution due to the small sample size (*n* = 60). However, these outcomes may provide information to help inform the design of the full-scale RCT by providing preliminary effect sizes and more accurate power calculations. In addition to the small sample size, other limitations include an unpredictable enrolment rate and loss to follow-up and the unknown ability of 3-D motion analysis to pick up the effects of AKP after tibial nailing. If there is substantial uncertainty and areas of concern about the feasibility of a future definitive RCT, then the protocol will be revised.

## Trial oversight

Oversight of the trial is the responsibility of the head of the Department for Orthopaedics and Trauma at the Royal Adelaide Hospital (who is independent of the trial team) and supported by the University of Adelaide’s Centre for Orthopaedic & Trauma Research. A Trial Steering Committee will be formed comprising of the chief investigator and associate investigator. A Data Safety and Monitoring Committee will be formed comprising an associate investigator, clinicians, and database management staff at the Royal Adelaide Hospital.

## Data Availability

De-identified motion capture data will be made available. This data does not contain video footage and can in no way be used to identify patients. The de-identified motion capture data will be made available on Figshare (online repository) with access subject to approvals by the Principal Investigator (A/Prof Mark Rickman: mark.rickman@sa.gov.au) to researchers who provide a methodologically sound proposal. Supplementary analytic code will also be made available.

## Abbreviations

IM: Intramedullary
RCT: Randomised controlled trial
IPN: Infrapatellar nailing
SPN: Suprapatellar nailing
MRI: Magnetic resonance imaging
PROMs: Patient-reported outcome measures
PBOMs: Performance-based outcome measures
3-D: Three-dimensional
NMS: New Mobility Score
KSS: Knee Society Score
KS-KS: Knee Society Knee Score
KS-FS: Knee Society Function Score
KOOS: Knee Injury and Osteoarthritis Outcome Score
KOOS-PF: KOOS Patellofemoral Subscale
MCID: Minimal Clinically Important Difference
FJS-12 Knee: Functional Joint Score-12 Knee
EQ-5D-5L: EuroQol 5-Dimension 5-Level
VAS: Visual analogue scale
PCS: Pain Catastrophizing Scale
AWT-K: Aberdeen Weight-Bearing Test (Knee)
ROM: Range of motion
MIQS: Maximum isometric quadriceps strength
TUG: Timed up-and-go
PCA: Principal component analysis
